# Effects of Fe and Mn cations on Cd uptake by rice plant in hydroponic culture experiment

**DOI:** 10.1371/journal.pone.0243174

**Published:** 2020-12-10

**Authors:** Babar Hussain, Jumei Li, Yibing Ma, Nazia Tahir, Aman Ullah

**Affiliations:** 1 Institute of Agricultural Resources and Regional Planning, Chinese Academy of Agricultural Sciences, Beijing, China; 2 Macao Environmental Research Institute, Macau University of Science and Technology, Macao, China; Government College University Faisalabad, PAKISTAN

## Abstract

Iron (Fe) and manganese (Mn) are nutritional components of rice, plays an important role in its physiological processes and can minimize absorption of cadmium (Cd) in rice. Fe, Mn, and Cd transporters such as *CAL1*, *OsNRAMP5*, *OsNRAMP1*, *OsIRT1*, *OsHMA3*, and *OsNAAT1* regulate uptake of Cd in rice. However, the effect of exogenous application of Fe, and Mn on the accumulation of Cd and relative expression (RE) of these transporters in rice has not been investigated. Therefore, a hydroponic culture experiment was conducted to investigate the impact of Fe and Mn on Cd uptake and RE of these transporters in rice. The results showed that the Fe and Mn application significantly decreased Cd in the roots and shoots of rice. Whereas, Cd concentration in the rice significantly increased with increasing Cd concentration in the solution. The addition of manganese in the culture medium can reduce the cadmium content of rice roots by 11.9–82.3% and shoots by 11.6–85.0%, while the addition of iron in the culture medium can reduce the cadmium content of rice roots and shoots by 26–65% and 9–683% respectively. Meanwhile, application of sufficient doses of Fe and Cd in solution culture increased RE of *CAL1*, *OsNRAMP5*, *OsNRAMP1*, *OsIRT1*, and *OsNAAT1* in roots, whereas expression level of *OsHMA3* was decreased. Similarly, expression level of *CAL1*, *OsNRAMP5*, and *OsNRAMP1* significantly increased in roots in high Cd and Mn deficient treatments. This may be concluded that the Cd increases expression of *CAL1*, *OsNRAMP5*, *OsNRAMP1*, *OsIRT1*, and *OsNAAT1* but decreases *OsHMA3* expression in rice roots, which resulted in increased Cd uptake in hydroponically grown rice.

## Introduction

Rice (*Oryza sativa* L.) is the staple food for more than half of the world’s population [1]. Cd pollution in rice has posed a serious threat to human health especially in Asian countries [2]. Cd can severely affect several human organs and systems such as the reproductive system, respiratory system, kidneys and skeletal system and can cause severe health problems such as Itai-Itai disease [2, 3]. Thus, minimizing Cd uptake by rice through fertilizer/nutrient management is an easy and effective method [4, 5].

Fe is an essential micronutrient and has effectively decreased Cd toxicity in different plant species [6–8]. Exogenous application of Fe can reduce Cd content in rice and improve rice growth and yield [8, 9]. Manganese is a key supplement for plant growth, which plays an important role in enzyme activation, biotic redox reactions, splitting of H_2_O, and decontamination O_2_ free radicals [10, 11]. Fe and Mn both alleviated Cd toxicity by reducing Cd accumulation and by upholding redox regulation that prevents Cd-inducible damage to root growth and photosynthesis [12]. Fe and Mn can form iron plaque on the surface of rice roots sequester Cd on roots [1, 13]. However, molecular studies showed that Cd translocation into rice occurs via Fe metabolic pathways which may be affected by Fe concentration in substrates [14, 15].

Rice accumulates more Cd than other cereal crops may be due to higher expression and functionality of the *OsNRAMP5* gene (responsible for Cd uptake by roots) [3]. Furthermore, several rice genes have been identified which take part in xylem loading and phloem redistribution of Fe and Cd at different locations in the rice plant. For example, *OsIRTI* and *OsNRAMP5* mediate uptake of Cd from the rhizosphere into root cells [15]. *OsHMA3* is involved in Cd compartmentalization into vacuoles in root cells [16]. It was found that the expression of *OsHMA3* was up-regulated under Cd stress in rice roots than that of control [17]. Several genes have been reported, which affects the uptake, transportation, and accumulation of Mn and Cd in rice. Cd from the root surface is primarily taken into root cells as a ‘hitchhiker’ via the Mn transporter *OsNRAMP5* [15]. *CAL1* (cadmium accumulation in leaf 1) mainly expressed in root exodermis and xylem parenchyma cells and sequesteres Cd in the cytosol, seems to minimize Cd content in cytosol there by carrying long-distance Cd transport through xylem vessels. *CAL1* did not show any effect on the accumulation of Cd in rice grain [18]. Thus, effect of exogenous application of Fe and Mn on the accumulation of Cd as well as relative expression of *CAL1*, *OsNRAMP5*, *OsNRAMP1*, *OsIRT1*, *OsNAAT1*, and *OsHMA3* in rice has not been investigated. Therefore, the present experiment was conducted to find out the impact of Fe and Mn cations on uptake of Cd in rice and expression level of aforementioned genes under combined application of Fe, Mn and Cd.

## Materials and methods

### Plant growth and treatments

A hydroponic pot experiment was conducted at greenhouse of the Chinese Academy of Agricultural Sciences Beijing (40° 0' 8.6364'' N, 116° 21' 57.5208'' E). The seeds of the “Huang Hua Zhan” rice variety, collected from Changsha city were surface sterilized for 15 minutes in 30% (v/v) H_2_O_2_ solution, thoroughly washed with deionized water and then soaked in distilled water in the dark for 48 hours. Rice seeds were then sandwiched into two filter papers that were placed vertically in petri dishes. The young seedlings were then transferred into beakers containing hydroponic solution for three weeks. After three weeks rice seedlings were transferred to pots for four weeks in a full-strength Hoagland nutrient solution (pH 5.5), consisting of 0.116 mg L^−1^ NH_4_NO_3_, 0.0499 mg L^−1^ NaH_2_PO_4_•2H_2_O, 0.087 mg L^−1^ K_2_SO_4_, 0.111 mg L^−1^ CaCl_2_, 0.418 mg L−1 MgSO_4_•7H_2_O, 0.091 mg L^−1^ (NH_4_)_6_MoO_24_•4H_2_O, 1.098 mg L^−1^ H_3_BO_3_, 0.0445 mg L^−1^ ZnSO_4_•7H_2_O, 0.0416 mg L^−1^ CuSO_4_•5H_2_O. The pH of the nutrient solution was set to 5.5 using morpholinoethanesulphonic acid (MES) and the solution was changed after every three days. In the full strength, Hoagland solution seedlings were treated with 0, 10, 20, and 30 mg L^−1^ Cd (supplied as CdSO_4_). While 0, 0.2, 0.4 and 0.6 mg L^−1^ Fe as FeSO_4_.7H_2_O with full-strength Hoagland solution were supplied for four weeks. The experiment had two factors, the first factor was Fe (Fe0, Fe0.2, Fe0.4 and Fe0.6) and the second was Cd (Cd0, Cd10, Cd20 and Cd30). The interactions of Fe and Cd combination resulted in 16 treatments, each replicated three times. The treatments combinations were Fe0Cd0 (no Fe and Cd applied), Fe0Cd10, Fe0Cd20, Fe0Cd30, Fe0.2Cd0, Fe0.2Cd10, Fe0.2Cd20, Fe0.2Cd30, Fe0.4Cd0, Fe0.4Cd10, Fe0.4Cd20, Fe0.4Cd30, Fe0.6Cd0, Fe0.6Cd10, Fe0.6Cd20 and Fe0.6Cd30. For Mn and Cd experiment the same procedure was followed. Cd was supplied as 0, 10, 20 and 30 mg L^−1^ Cd (CdSO4), while Mn treatments were as 0, 0.5, 1.0, and 2.5 in the form of MnSO4. Here the two factors were Mn (Mn0, Mn0.5, Mn1 and Mn1.5) and Cd (Cd0, Cd10, Cd20 and Cd30). Thus, interaction of Mn and Cd treatments also resulted in 16 combination as follows: Mn0Cd0 (no Mn and Cd supply), Mn0Cd10, Mn0Cd20, Mn0Cd30, Mn0.5Cd0, Mn0.5Cd10, Mn0.5Cd20, Mn0.5Cd30, Mn1.0Cd0, Mn1.0Cd10, Mn1.0Cd20, Mn1.0Cd30, Mn2.5Cd0, Mn2.5Cd10, Mn2.5Cd20, and Mn2.5Cd30. The experiment was carried out in a greenhouse with 70% humidity, the temperature varying between 25–35°C, with light exposure of 12–14 h d^−1^. The photographs of the experiment are shown in S1 Fig.

### Chemical analysis of roots and leaves

The rice roots and leaves were harvested at the end of the experiment, cleaned with tap water followed by a wash with distilled water In order to achieve a constant weight, samples were oven-dried at 100°C. A stainless steel grinder was used to crush dried rice plant materials (i.e. roots and shoots) into fine powder and accurately weighed (0.5 g) into clean, dry digestion tubes (100 ml). Then 6 ml of concentrated nitric acid (HNO_3_) and 3 ml of hydrogen peroxide (H_2_O_2_) were added and left overnight in the digestion tubes. The tubes were then placed in a high-pressure sealed digestion microwave where the temperature was set at 200°C and kept for two hours. The tubes were put on a heating block after digestion in the microwave and kept for around two to three hours. The solutions were then cooled at room temperature, diluted to 50 ml with ultra-pure water containing 5% HNO3; the samples were gently shaken and then filtered [19]. The total Fe, Mn Subsequently and Cd in digested solutions were measured by inductively coupled plasma mass spectrometry (ICP-MS).

### RNA extraction and QRT-PCR

The total RNA was obtained from four weeks old seedlings. Samples of fresh roots and shoots were collected using Trizol reagent (Life, Japan). For each sample, approximately 2μ of RNA was used for reverse transcription with a PrimerScript 1st strand cDNA synthesis Kit (Takara, Japan). The QRT-PCR assays were carried out using SYBR Premix Ex Taq (Takara, Dalian, China) on the AB17500 PCR system (Life Technologies, USA). The expression levels of target genes were normalized to that of OsActin. All QRT-PCR assays were performed in three independent replications. Relative gene expression levels were detected using the 2^–ΔΔCT^ method [20]. The primers used in this study are listed in Table 1.

**Table 1:** Primers used for relative genetic expression.

Primer code	Base sequence (5' to 3')
*CAL1-Q-*F	AGTCGCGTGTTCTCCTTTGT
*CAL1-Q-*R	CATGACAGCAGCTTGCAAAT
*OsNAAT1-Q-*F	GGAGGGAATCCATGATGATG
*OsNAAT1-Q-*R	GGCAGAAGGATTTGATCCTCTC
*OsIRT1-Q-*F	GAACCGCGTCGTCGTTCAG
*OsIRT1-Q-*R	CCATCCCCTCGAACATCTGG
*OsIRT2-Q-*F	TCATGCTCACGTTCCACACG
*OsIRT2-Q-*R	GAGAACCTGCACAATGACGC
*OsNramp1-Q-*F	TCTCTGTCTCCGGCACTGTA
*OsNramp1-Q-*R	CATCAGGTTCCGAAGCCACT
*OsNramp5-Q-*F	GAAGTGGCTTCGGAACCTGA
*OsNramp5-Q-*R	GAAGCTCGTGCTCAGGAAGT
*OsHMA2-Q-*F	GAGGGAGGGAGGTGTCAGAA
*OsHMA2-Q-*R	TGGTGATCTTCTCACTGCCG
*OsHMA3-Q-*F	AGAACAGCAGGTCGAAGACG
*OsHMA3-Q-*R	ATTGCTCAAGGCCATCTGCT
*OsLCT1-Q-*F	GAGTTCTTCGTCAGAGCTAC
*OsLCT1-Q-*R	CAGTGCTGGATGACGAATTG

### Quality control

To ensure the accuracy of the results, a standard reference material (GBW08513) from the National Research Center for Standards of China was used. The metals recovery rate ranged from 95% to 104%.

### Statistical analysis

Descriptive statistics were conducted by Microsoft Excel 2016. Analysis of variance (ANOVA) was conducted by XLSTAT software [21]. Mean data was tested by Duncan’s multiple range test at (p < 0.05), conducted with the XLSTAT.

## Results

### Effect of Fe, Mn and Cd on biomass of rice roots and shoots

**Fig 1:**
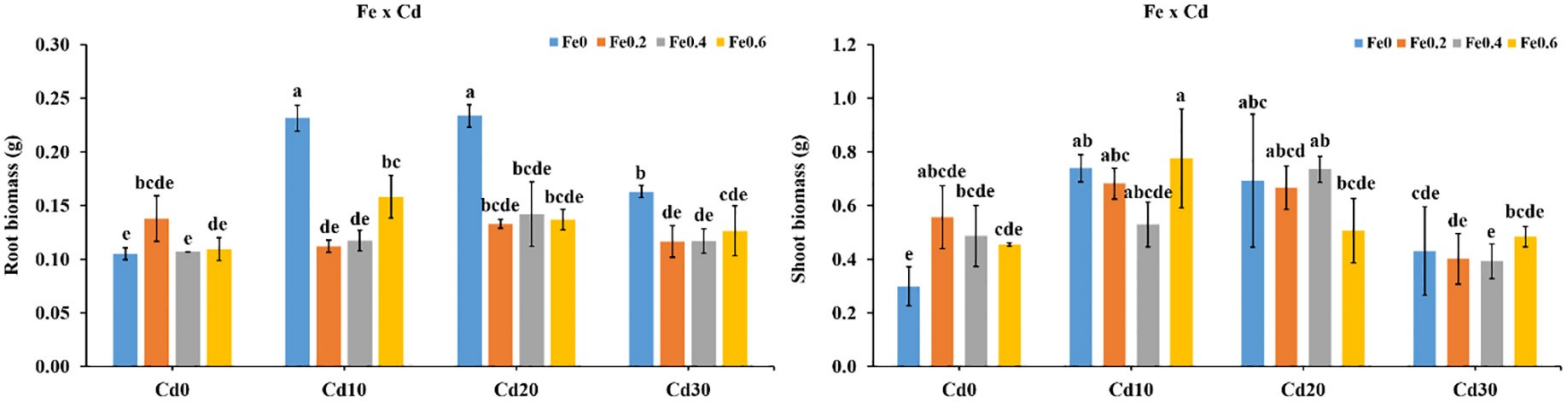
Effect of Fe and Cd interaction on root and shoot biomass (g). Here, Fe and Cd indicates 0, 0.2, 0.4 and 0.6 mg L^−1^ Fe as FeSO_4_.7H_2_O and 0, 10, 20 and 30 mg L^−1^ Cd (supplied as CdSO_4_). The data presented are mean ± standard deviation (n = 3). Mean values followed by different letter are significantly different using Duncan’s multiple range test at 5% level.

**Fig 2:**
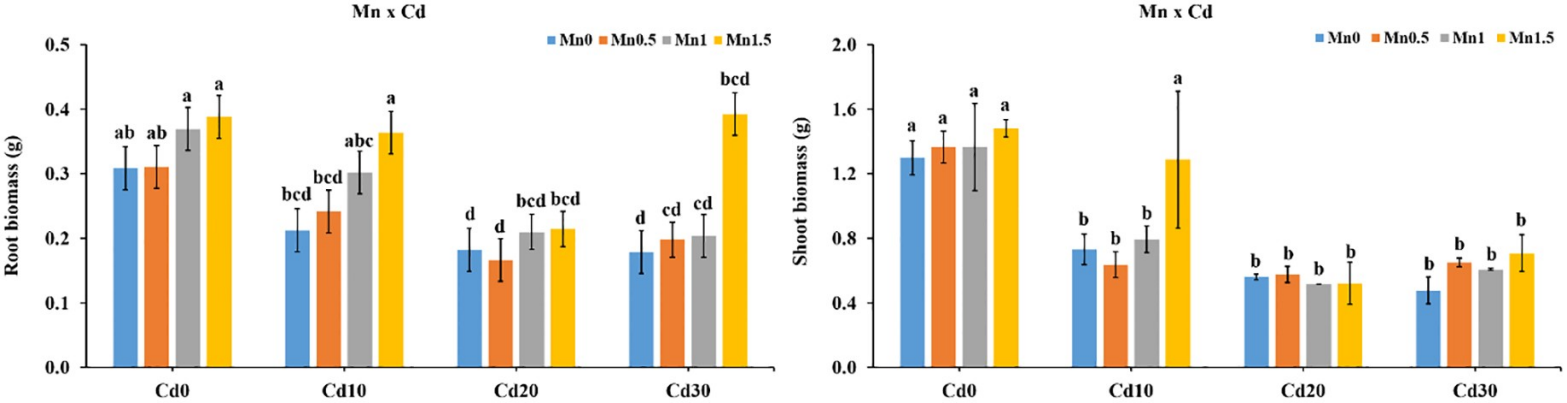
Effect of Mn and Cd interaction on root and shoot biomass (g). Here, Mn and Cd indicates 0, 0.5, 1 and 1.5 mg L^−1^ Mn as MnSO4 and 0, 10, 20 and 30 mg L^−1^ Cd (supplied as CdSO_4_).

Results showed that the biomass of roots and shoots increased under high Fe and low Cd levels (Fig 1). Relative to Fe0Cd0, the roots biomass was increased by 31.4% in Fe0.2Cd0 while average increased 67.3% was recorded from Fe0.2Cd0, Fe0.4Cd0, and Fe0.6Cd0 treatments. However, it can be noted that the high doses of Cd combine with Fe also increased roots and shoots biomass. Application of Mn significantly promoted crop growth. Roots and shoots biomass was increased with increasing Mn concentration in the solution. The roots and shoots biomass increased by 22% and 12.7% on average in Mn0.5Cd0, Mn1Cd0 and Mn1.5Cd0 respectively as compared to Mn0Cd0 treatment (Fig 2).

### Effect of Fe on Cd uptake by rice root and shoot

**Fig 3:**
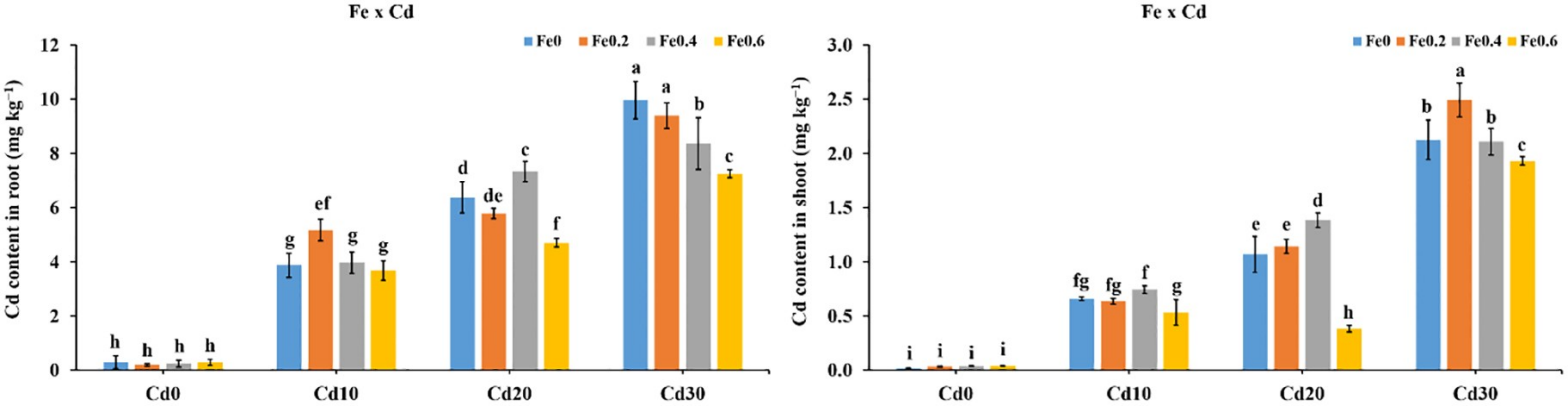
Effect of Fe and Cd interaction on Cd accumulation in rice root and shoot.

Roots and shoots Cd concentration was ranged from 0.20–9.96 mg kg^−1^ and 0.02–2.49 mg kg^−1^ respectively. At the low Cd level treatment, the Cd in roots and shoots decreased with increase in Fe concentration. The lower Cd in roots were found in Fe0Cd0, Fe0.2Cd0, Fe0.4Cd0, and Fe0.6Cd0 with an average value of 0.29 mg kg^−1^. The highest Cd 9.96 mgkg^−1^ in the roots was found in Fe0Cd30 treatment (Fig 3). Moreover, Cd concentrations in shoots were lower in Fe0Cd0, Fe0.2Cd0, Fe0.4Cd0, and Fe0.6Cd0 as compared to other treatments. The highest total Cd content in shoot was found in Fe0.2Cd30 treatment 2.49 mg kg^−1^ followed by Fe0Cd30, Fe0.4Cd30 and Fe0.6Cd30 than Fe0Cd0 (Fig 3).

### Effect of Mn on Cd uptake by rice roots and shoots

In Mn0Cd0, Mn0Cd10, Mn0Cd20 and Mn0Cd30 treatments, the total Cd content in roots were 0.13, 3.09, 7.54, 9.78 mg kg^−1^ respectively, while the total Cd concentration in shoots were 0.03, 0.74, 1.29, 2.27 mg kg^−1^ respectively. Under the low Cd level treatments, the Cd content of roots and shoots decreased with the increase in Mn concentration in solution. The range of total Cd reduce ratio was 11.9–82.3% in roots and 11.6–85.0% in shoots respectively.

**Fig 4:**
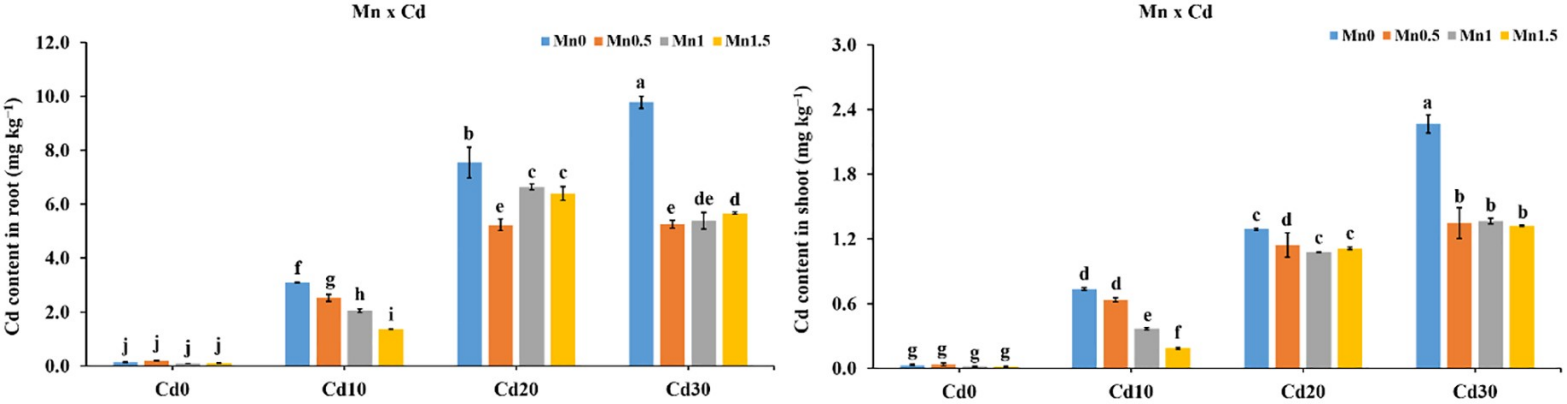
Effect of Mn and Cd interaction on Cd accumulation in roots and shoots of rice.

The concentration of Cd in roots and shoots under Mn0.5Cd0, Mn1Cd0 and Mn1.5Cd0 treatments was non-significantly different than Mn0Cd0. While, in Mn0Cd10, Mn0Cd20 and Mn0Cd30 treatments, the average Cd content was 49.9 and 44.3 times higher than Mn0Cd0 in roots and shoots respectively. Overall, the lowest Cd contents in the roots were found in plants treated with Mn1.0Cd0, Mn0.5Cd0, Fe0Cd0 and Mn1.5Cd0, while the highest Cd content in roots was found in Mn0Cd30 treatment. In the shoots the concentration of Cd was found lowest in Mn1.5Cd0, Mn1Cd0, Fe0Cd0 and Mn0.5Cd0, and greatest Cd content was found in Mn0Cd30 with the value of 2.27 mg kg^−1^ (Fig 4).

### Genes expression in rice roots and shoots under Fe and Cd addition

**Fig 5:**
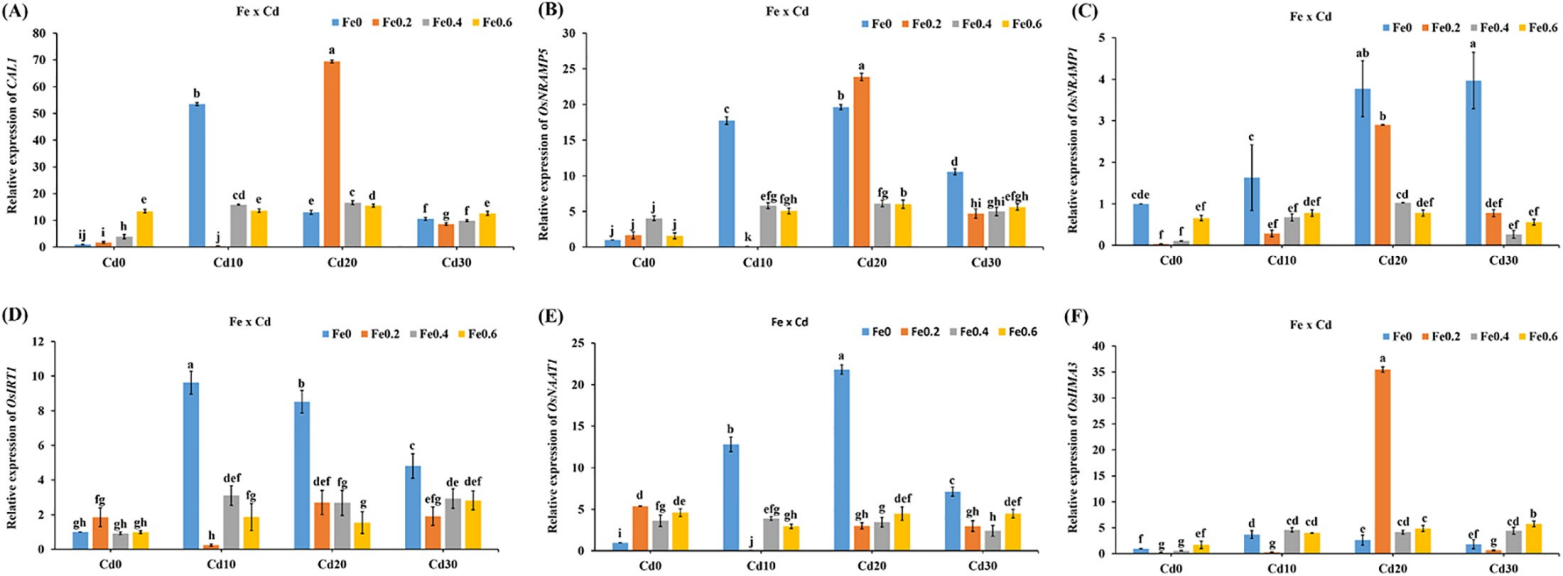
Effect of Fe and Cd interaction on relative genetic expression of *CAL1*, *OsNRAMP5*, *OsNRAMP1*, *OsIRT1*, *OsNAAT1*, and *OsHMA3* in rice roots.

**Fig 6:**
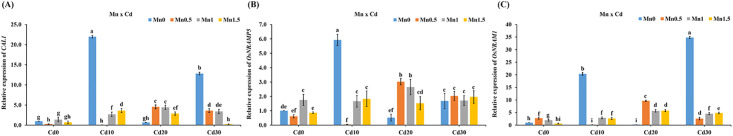
Effect of Mn and Cd interaction on relative genetic expression of *CAL1*, *OsNRAMP5*, and *OsNRAMP1* in rice roots.

*CAL1*, *OsNRAMP5*, *OsNRAMP1*, *OsIRT1*, *OsNAAT1*, and *OsHMA3* are considered as important Cd transporters. The results showed that the different levels of Fe and Cd in the solution had a substantial effect on the level of expression of genes in both rice roots and shoots. In the roots *CAL1* expression increased significantly by 0.72–12.4 times in the Fe0.2Cd0, Fe0.4Cd0, and Fe0.4Cd0 treatments relative to Fe0Cd0 (Fig 5A). While the expression of *CAL1* in the Fe0Cd10, Fe0Cd20, and Fe0Cd30 treatments increased by 9.5–52.5 times than Fe0Cd0. Whereas, in Fe0.2Cd20, Fe0.2Cd30, Fe0.4Cd10, Fe0.4Cd20, Fe0.4Cd30, Fe0.6Cd10, Fe0.6Cd20, and Fe0.6Cd30 the *CAL1* expression in roots increased by 7.6–68.4 times. *OsNRAMP5* and *OsNRAMP1* are crucial transporters of Fe and Cd that carry Fe and Cd from the surface of the root into root cells. *OsNRAMP5* expression increased by 0.5–3.0 times in roots treatments under Fe0.2Cd0, Fe0.4Cd0, and Fe0.6Cd0 relative to Fe0Cd0. The relative expression of *OsNRAMP5* also increased in the roots by 9.5–18.6 times in the Fe0Cd10, Fe0Cd20, and Fe0Cd30 treatments. Under Fe0.2Cd20, Fe0.2Cd30, Fe0.4Cd10, Fe0.4Cd20, Fe0.4Cd30, Fe0.6Cd10, Fe0.6Cd20, and Fe0.6Cd30 treatments, the expression level of OsNRAP5 increased by 3.6–22.8 times in roots compared with Fe0Cd0 (Fig 5B). The expression of *OsNRAMP1* in roots decreased by 34.2%-96.5% in Fe0.2Cd0, Fe0.4Cd0, and Fe0.4Cd0 treatments whereas, it increased by 63.2%-296% under Fe0Cd10, Fe0Cd20, and Fe0Cd30 treatments. The expression of *OsNRAMP1* also decreased by 22.1%-73.2% in Fe0.2Cd10, Fe0.2Cd30, Fe0.4Cd20, Fe0.4Cd30, Fe0.6Cd10, Fe0.6Cd20, and Fe0.6Cd30 treatments than Fe0Cd0 (Fig 6C). While relative expression of *OsNRAMP1* enhanced by 190% and 2.85% under Fe0.2Cd20 and Fe0.2Cd40 treatments respectively (Fig 5C). The relative expression of *OsIRTI* in the roots decreased by 7.65% and 1% in the Fe0.4Cd0, and Fe0.6Cd0 treatments respectively, while it increased by 84.6% under Fe0.2Cd0 treatment as compared to Fe0Cd0 treatment. However, the expression increased by 3.8–8.6 times in Fe absence and Cd sufficient treatments such as Fe0Cd10, Fe0Cd20, and Fe0Cd30. In the Fe and Cd sufficient treatments (Fe0.2Cd20, Fe0.2Cd30, Fe0.4Cd10, Fe0.4Cd20, Fe0.4Cd30, Fe0.6Cd10, Fe0.6Cd20, and Fe0.6Cd30) the expression of *OsIRT1* in roots enhanced by 54.2%-210% (Fig 5D). Compared to Fe0Cd0, in the Fe0.2Cd0, Fe0.4Cd0, and Fe0.6Cd0 treatments, the relative expression of *OsNAAT1* increased by 2.6–4.4 times. Whereas, the expression values for *OsNAAT1* were 6.1–20.7 times higher in the Fe0Cd10, Fe0Cd20, and Fe0Cd30 treatments than Fe0Cd0. In the Fe0.2Cd20, Fe0.2Cd30, Fe0.4Cd10, Fe0.4Cd20, Fe0.4Cd30, FE0.6Cd10, Fe0.6Cd20, and Fe0.6Cd30 treatments the expression of *OsNAAT1* increased by 141%-348% (Fig 5E). The relative expression of *OsHMA3* reduced by 81.5% and 41.6% in the Fe0.2Cd0, Fe0.4Cd20 treatments respectively, while the relative expression of *OsHMA3* enhanced by 71.2% in Fe0.6Cd0 treatment as compared to Fe0Cd0. The *OsHMA3* expression level also decreased by 72.8% and 33.1% under Fe0.2Cd10 and Fe0.2Cd30 treatments respectively (Fig 5F). The greatest expression of *OsHMA3* was recorded under the Fe0.2Cd20 treatment and it was enhanced by 34.4 times. Under the treatments Fe0.4Cd10, Fe0.4Cd20, Fe0.4Cd30, Fe0.6Cd10, Fe0.6Cd20, and Fe0.6Cd30 the *OsHMA3* expression increased by 0.7–4.74 times in roots than that of Fe0Cd0.

### Genes expression in rice roots and shoots under Mn and Cd application

The application of Mn and Cd in the nutrient solution showed a significant impact on the expression levels of *CAL1*, *OsNRAMP5* and *OsNRAMP1* in roots. The relative expression of *CAL1* in the roots was reduced by 718%, 29.6%, and 32.1% under Mn0.5Cd0, Mn1.5Cd0 and Mn0Cd20 treatments respectively as compared to Fe0Cd0 (Fig 6A). In the Mn0Cd10 and Mn0Cd30 treatments gene expression was increased by 2 and 1.1 fold respectively as relative to Mn0Cd0. Similarly, under the Mn0.5Cd20, Mn0.5Cd30, Mn1Cd10, Mn1Cd20, Mn1Cd30, Mn1.5Cd10, and Mn1.5Cd20 treatments the expression of *CAL1* also enhanced by 169%-357% as compared to Mn0Cd0, *OsNRAMP5* expression decreased by 38.4%, 13.1% and 47.2% respectively under the treatments Mn0.5Cd0, Mn1.5Cd0 and Mn0Cd20, whereas it was increased by 492% and 68.3% respectively in the treatments Mn0Cd10 and Mn0Cd30. The genes expression of *OsNRAMP5* was also increased by 52.7%-202% in Mn0.5Cd20, Mn0.5Cd30, Mn1Cd10, Mn1Cd20, Mn1Cd30, Mn1.5Cd10, Mn1.5Cd20, and Mn1.5Cd30 treatments (Fig 6B). The expression of *OsNRAMP1* in Mn0.5Cd0 and Mn1Cd0 treatments enhanced by 177% and 97.6% respectively. Although *OsNRAMP1* expression was decreased by 33.4%, 84.5% and 78.5% in Mn1.5Cd0, Mn0.5Cd10, and Mn0Cd20 treatments respectively. Whereas, its expression was enhanced by 159%-868% in Mn0.5Cd20, Mn0.5Cd30, Mn1Cd10, Mn1Cd20, Mn1Cd30, Mn1.5Cd10, Mn1.5Cd20, and Mn1.5Cd30 treatments (Fig 6C).

## Discussion

Compared to Fe0Cd0 treatment, the roots biomass was increased by 31.4% in Fe0.2Cd0 treatment, while shoots biomass in Fe0.2Cd0, Fe0.4Cd0, and Fe0.6Cd0 was 67.3% higher than Fe0Cd0 on average (Fig 1). Similarly, roots and shoots biomass was increased in Mn0.5Cd0, Mn1Cd0 and Mn1.5Cd0 treatments containing excessive Mn and Cd deficient doses (Fig 2). Adhikari [22], found that supply of both Fe and Cd in hydroponic experiment significantly affected plant growth and yield, as well as accumulation of Cd in plant tissues. Furthermore, it was confirmed that the dry matter production of rice shoot was highest at the highest activity level of Fe [22]. It was found that the addition of Fe in the soil increased root and shoot dry weight of rice [8]. Moreover, it has been reported that the exogenous application of Mn at the rate from 0.05 μM to 800 μM under hydroponic experiment can reduce accumulation of Cd in under and above ground plant parts in rice [23]. However, increasing Cd concentration in solution has reduced roots and shoots biomass under Fe and Mn addition (Figs 1 and 2). It was confirmed that the plant growth was inhibited under Cd stress in four rice cultivars as compared to normal conditions [24]. Root growth was limited and number and length of roots and tillers was also decreased by Cd in the rice cultivars [24].

At the low Cd level treatments, the Cd in roots and shoots decreases with increase in Fe concentration. Such as Cd concentrations in shoots were lower in Fe0Cd0, Fe0.2Cd0, Fe0.4Cd0, and Fe0.6Cd0 as compared to other treatments (Fig 3). It is in consistence with that Fe and Mn could decrease Cd uptake and minimize Cd inducible rhizotoxicity [12]. Exogenous Fe application can significantly decrease Cd concentration in rice roots and shoots [25]. It was further confirmed that Fe^2+^ cations could compete with Cd^2+^ ions for adsorption sites at the roots surface and as a result uptake of Cd in rice is decreased [26]. Consistence with our results [27, 28], it was observed that under a Fe-sufficient supply, Cd concentration in rice stem and leaves was reduced. Fe fertilization directly and effectively increased Fe content and decreased Cd contamination to some extent. The reason may be Fe^2+^ competes with Cd for the same binding sites and follow similar transport pathways on the surface of roots cells [29]. Also, Fe oxides have a significant ability for Cd adsorption and can effectively immobilize Cd [30]. However, the highest Cd concentration of 9.96 mg kg^−1^ in the roots was found in Fe0Cd30 treatment (Fig 3). The highest total Cd content in shoot was found in Fe0.2Cd30 treatment 2.49 mg kg^−1^ followed by Fe0Cd30, Fe0.4Cd30 and Fe0.6Cd30 than Fe0Cd0 (Fig 3). It was reported that Cd content in DCB extracts enhanced with increasing Cd and Fe addition [25]. Furthermore, roots and shoots Cd concentration enhanced with enhancing Cd supply [25]. It was further reported that FeSO_4_ fertilizer significantly enhanced cadmium content in roots as well as shoots of rice than CK [31]. The reason was may be due to the Fe transporter *OsIRT1* in the cell membrane which mainly expressed in rice roots directly carrying Fe^2+^ via cell membrane and Cd^2+^ as well [31–33]. A similar finding was found by [34], which stated that Cd^2+^ bound to the apoplastic membrane and remains in the root cell wall after desorption while saturated Cd^2+^ from the solution influx across the root cell plasma membrane was mediated by the transporter. Furthermore, Fe deficiency induces the expression of IRTI in *Arabidopsis* which may assisted translocation of Cd^2+^ [34].

The Cd content of the roots and shoots decreased with increase of Mn concentration in solution under the low Cd level treatments. The range of total Cd reduction was 11.9–82.3% in roots and 11.6–85.0% in shoots. In the shoots the concentration of Cd was found the lowest in Mn1.5Cd0, Mn1Cd0, Fe0Cd0 and Mn0.5Cd0 (Fig 4). Qin [35], noted that rice plant height was significantly increased and decreased Cd toxicity to normal level under Mn dose of 0.5 mg L^−1^. The roots Cd content in two rice genotypes were significantly reduced by the increased MnSO_4_ and EDTA•Na_2_Mn application in solution than CK [11]. The findings are in agreement with that the application of 40 μM Fe and 2 μM Mn increased biomass and reduced Cd concentration in rice under Cd stress of 5 and 25 mM. Furthermore, it was concluded that Fe and Mn alleviated Cd toxicity by decreasing Cd content in rice and by regulating redox potential which hinders damage to root growth and photosynthesis caused by Cd [12]. Moreover, application of 0.3 mM MnSO_4_ in hydroponic solution reduced cadmium absorption in roots and shoots by 40% and 60% respectively in rice seedlings [36]. Because, Mn is a divalent cation that is absorbed by plants through an active transport mechanism which comes in competition with Cd^2+^ as Mn and Cd contains similar pathways for plant transport and accumulation [36]. While, in Mn0Cd10, Mn0Cd20 and Mn0Cd30 treatments, the average Cd content was 49.9 and 44.3 times higher than Mn0Cd0 in roots and shoots respectively. Moreover, the highest Cd content in the shoot was found in Mn0Cd30 treatment with the value of 2.27 mg kg^−1^ (Fig 4). It was confirmed that Cd accumulation in roots was directly associated with the Cd content in solution [22]. Therefore, two possible mechanisms may exist for the accumulation of Cd in combination with Fe and Mn. First may be due to the desorption of Cd^2+^ ions from the root cell wall and then mediated by Cd transporters IRTI [34], such as *OsIRT1* and other transporters in our experiment. Secondly, various studies reported that the formation of Fe and Mn plagues on the roots had no significant effect on the uptake of Cd in plants [37–39]. Huang [39] noted that *Phytolacca acinosa* Roxb (*P*. *acinosa*) plants treated with 50 mg L^−1^ Cd accumulated higher level of Cd as compared to plants exposed to 2 mg L^−1^ Cd, especially in the plague treatments (p < 0.05). Moreover, it was reported that DCB extractable Cd in the roots and shoots of *Kandalar*. *Obovata* (S.L.) significantly enhanced with an increasing Cd supplementation [38]. Thus, the results may depend on several factors such as amount of metal plaque, the concentration of metal and the pH in the culture solution [13, 38, 39].

Relative expression of genes were significantly affected by Fe and Cd in the solution. The expression of *CAL1* enhanced by 0.7–12.4 times under the Fe0Cd10, Fe0Cd20, and Fe0Cd30 treatments than Fe0Cd0. Whereas, in the Fe0.2Cd20, Fe0.2Cd30, Fe0.4Cd10, Fe0.4Cd20, Fe0.4Cd30, Fe0.6Cd10, Fe0.6Cd20, and Fe0.6Cd30 treated plants the *CAL1* expression in roots increased by 7.6–68.4 times (Fig 5A). Luo [18] found *CAL1* is a defensin-like protein and localized in root exodermis and xylem parenchyma cells. *CAL1* expression in the roots was induced by exposure to Cd, with the near-isogenic line (NIL) containing the TN1 allele showing a greater response to Cd than the NIL(CJ06) control line [18]. In addition, it was reported that *CAL1* was preferentially expressed in root and leaf sheath of rice seedlings. It was further confirmed that expression of *CAL1* was significantly induced under Cd exposure in near-isogenic line NIL(TNI) in root and expression in various tissues except for leaf blades [18]. The expression of *OsNRAMP5* enhanced by 0.5–3.0 times under Fe0.2Cd0, Fe0.4Cd0, and Fe0.6Cd0 treatments in roots than Fe0Cd0 treatment. While, in the Fe0Cd10, Fe0Cd20, and Fe0Cd30 treatment the relative expression of *OsNRAMP5* also increased by 9.5–18.6 times in roots. The expression level of *OsNRAMP5* enhanced by 3.6–22.8 times in roots under Fe0.2Cd20, Fe0.2Cd30, Fe0.4Cd10, Fe0.4Cd20, Fe0.4Cd30, Fe0.6Cd10, Fe0.6Cd20, and Fe0.6Cd30 treatments than Fe0Cd0 (Fig 5B). These results suggest that a synergistic effect may have existed between Cd concentration in solution and relative expression of *OsNRAMP5*. The results are in consistent that gene expression *OsNRAMP5* in roots was up-regulated under two doses of Cd treatments (1 μmol L^−1^ and 5 μmol L^−1^ Cd) [40]. Meanwhile, *OsNRAMP1* expression level reduced by 34.2%-96.5% in Fe0.2Cd0, Fe0.4Cd0, and Fe0.4Cd0 treatments whereas, increased by 63.2%-296% under Fe0Cd10, Fe0Cd20, and Fe0Cd30 treatments in roots (Fig 5C). The results are in accordance with that the expression of *OsNRAMP* which was induced by Cd treatment or by Fe inadequacy in both roots and shoots [41]. Overexpression of *OsNRAMP1* in rice roots enhanced the accumulation of Cd in leaves [14]. Cd accumulation in the shoot of rice increased due to higher expression of *OsNRAMP1*, this showed that *OsNRAMP1* could uptake and transport Cd in addition to Fe [42]. It was confirmed that among the seven members of the NRAMP family in rice, expression of *OsNRAMP1* was increased in roots and shoots under Cd stress, whereas on contrary our results *OsNRAMP5* (Os07g0257200) expression was decreased in both tissues in presence of Cd [43]. It was further confirmed that the expression of *OsNRAMP1* in yeast cells significantly increased the accumulation of Cd (5-fold) than that of control [44]. The relative expression of *OsIRTI* in Fe0Cd10, Fe0Cd20, and Fe0Cd30 treatments increased by 3.8–8.6 times. In the Fe and Cd sufficient treatments (Fe0.2Cd20, Fe0.2Cd30, Fe0.4Cd10, Fe0.4Cd20, Fe0.4Cd30, Fe0.6Cd10, Fe0.6Cd20, and Fe0.6Cd30) the expression of *OsIRT1* in roots increased by 54.2%-210% (Fig 5D). It has been documented that various transporters regulated Fe transportation in IRT and NRAMP families involved in Cd transport by rice [14, 45]. It was confirmed that Fe(II) mediated transporter such as *OsIRT1* and *OsIRT2* take part in Cd uptake in Fe-deficient rice grown in hydroponic culture [33]. The expression of *OsIRT1* was found higher in roots and was up-regulated under lower Fe conditions [46]. In addition to Fe and Cd also enters into the root cell through *OsIRT1* [33]. Compared to Fe0Cd0, the relative expression of *OsNAAT1* in the Fe0Cd10, Fe0Cd20, and Fe0Cd30 enhanced by 6.1–20.7 times. Whereas, in the Fe0.2Cd20, Fe0.2Cd30, Fe0.4Cd10, Fe0.4Cd20, Fe0.4Cd30, FE0.6Cd10, Fe0.6Cd20, and Fe0.6Cd30 treatments *OsNAAT1* expression increased by 141%-348% (Fig 5E). Similar findings have been revealed by [47] which states that *OsNAAT1* was highly up-regulated under Fe deficiency and Cd stress both in roots and shoots [47]. Furthermore, it was confirmed that when 1 mM Cd was added to the nutrient solution, Cd concentration in both naat1 roots or shoots was about 50% higher than that those in wild-type seedlings [48]. The relative expression of *OsHMA3* reduced by 81.5% and 41.6% in Fe0.2Cd0, Fe0.4Cd20 treatments respectively, while the relative expression of *OsHMA3* enhanced by 71.2% in Fe0.6Cd0 treatment as compared to Fe0Cd0. Whereas, its expression was also decreased by 72.8% and 33.1% under Fe0.2Cd10 and Fe0.2Cd30 treatments respectively (Fig 6F). Under treatments Fe0.4Cd10, Fe0.4Cd20, Fe0.4Cd30, Fe0.6Cd10, Fe0.6Cd20, and Fe0.6Cd30 treatments the *OsHMA3* expression was increased by 297%-474% than that of Fe0Cd0. It was confirmed that the application of Fe chelates in hydroponic culture did not significantly affect *OsHMA3* expression [28]. The results are in accordance with [49], who stated the higher expression of *OsHMA3* in the root. Furthermore it was confirmed that expression of *OsHMA3* was significantly increased in excessive Fe treatment [27]. Lu [50] reported, that overexpression of *OsHMA3* significantly reduced Cd translocation from roots to shoots and enhanced Cd tolerance. When Cd enters into the cytosol, *OsHMA3* sequestered Cd into vacuole [51]. However, [49] discovered an allele of *OsHMA3* which failed to transport Cd into vacuole in high Cd accumulating cultivars. Therefore in the present experiment, it may be possible that *OsHMA3* failed to sequester Cd in a vacuole. That’s why accumulation of Cd was found higher in Fe0.2Cd30, Fe0Cd30, Fe0.4Cd30 and Fe0.6Cd30 treatments. Furthermore, it was confirmed that *OsHMA3* expression was directly proportional to Cd concentration in the medium [52].

The application of Mn and Cd in the nutrient solution showed a significant impact on expression levels of *CAL1*, *OsNRAMP5* and *OsNRAMP1*. Compared to Mn0Cd0 treatment, the relative expression of *CAL1* in the Mn0Cd10 and Mn0Cd30 treatments increased by 20.9 and 11.8 times. Meanwhile, the expression of *CAL1* under the Mn0.5Cd20, Mn0.5Cd30, Mn1Cd10, Mn1Cd20, Mn1Cd30, Mn1.5Cd10, and Mn1.5Cd20 treatments also enhanced by 169%-357% (Fig 6A). It was confirmed that *CAL1* expression in the roots was significantly induced by exposure to Cd [18]. The results are in accordance with that expression of *CAL1* was also found high in various tissues except for leaf blades while in node I, and the adjoining flag leaf sheath its expression was significantly higher. In addition, higher Cd concentration was observed in seedling leaf blade and mature plant straws. Thus, it was suggested that *CAL1* might specifically carry Cd from roots to shoots, but not from shoot to grain [18]. This shows that the application of Mn significantly affects *CAL1* expression, however we don’t know the reason behind this. Therefore, further trials may be conducted to know the mechanism behind it.

Compared to Mn0Cd0, the expression of *OsNRAMP5* increased by 492% and 68.3% in Mn0Cd10 and Mn0Cd30 treatments respectively. The expression of *OsNRAMP5* also increased by 52.7%-202% in under Mn0.5Cd20, Mn0.5Cd30, Mn1Cd10, Mn1Cd20, Mn1Cd30, Mn1.5Cd10, Mn1.5Cd20, and Mn1.5Cd30 treatments (Fig 6B). It was confirmed that the deficiency of Fe^2+^ and M^2+^ induced the up-regulation of *OsNRAMP5* [53]. However, [43] reported that *OsNRAMP5* was not up-regulated under Mn deficiency. While, a recent study showed that the expression of *OsNRAMP5* was remarkably enhanced 2.33–5.67 folds in roots of three rice genotypes at Mn phytotoxicity condition than that of Mn deficient condition [54]. Thus, our experiment showed that both Mn and Cd affects the expression of *OsNRAMP5*. Therefore, further studies can be carried out to find the mechanism behind. It was suggested that the fluctuation of *OsNRAMP5* expression level may be different among rice genotypes and different hydroponic solution systems [54]. As the *OsNRAMP1* expression enhanced by 159%-868% in Mn0.5Cd20, Mn0.5Cd30, Mn1Cd10, Mn1Cd20, Mn1Cd30, Mn1.5Cd10, Mn1.5Cd20, and Mn1.5Cd30 treatments than Mn0Cd0 (Fig 6C). Previous studies confirmed that the expression of *OsNRAMP1* was up-regulated under Fe deficiency in rice [42] and Mn deficiency in Arabidopsis [55]. Similarly, [43] reported that expression of *OsNRAMP1* in roots and shoots of rice increased during Cd exposure. Thus the present experiment indicated that the expression of *OsNRAMP1* was affected by both Mn and Cd cations. In a recent study it has been showed that *OsNRAMP1* was predominantly expressed in root plus leaf as well as fixed plasma film restricted protein [41]. *OsNRAMP1* articulation was instigated by exposure to Cd and Fe inadequacy. Immunostaining demonstrated that *OsNRAMP1* confined in all root cell excluding central vasculature, and in leaf mesophyll cell [41]. The knockout of *OsNRAMP1* brought about noteworthy reductions in root take-up of Cd and Mn and their amassing in rice shoot and grain, and expanded affectability to Mn insufficiency. The knockout of *OsNRAMP1* showed less effect on Cd and Mn take-up compared to knockout of *OsNRAMP5*, while knockout of the two qualities brought about enormous declines in the take-up of the Cd and Mn. While, *OsNRAMP1* plays an essential role in take-up of manganese and cadmium in rice and the role of *OsNARAMP5* and *OsNRAMP1* are comparative yet not excess [41]. Thus it can be concluded that in the present experiment, variations in expression of all the genes may also be affected by the rice variety and hydroponic solution.

## Conclusions

This study demonstrated the expression levels of cadmium-related genes in rice under conditions of cadmium pollution and their relationship with iron and manganese nutrition were ascertained. It may be concluded that the application of Fe and Mn cations in rice under Cd exposure significantly decreased Cd concentration in roots and shoots. Increasing level of Cd in solution significantly increased Cd concentration in rice roots and shoots. Expression level of *CAL1*, *OsNRAMP5*, *OsNRAMP1*, *OsIRT1*, and *OsNAAT1* were increased with sufficient Cd and Fe and/or Mn insufficient treatments while *OsHMA3* expression decreased. In order to find the Fe and Mn effects on Cd, further research should be conducted under different conditions and under different doses of Fe and Mn and the deep insight process involved during Cd absorption and transportation.

## Supporting information

S1 Fig Experimental photograph. Fe and Cd indicates 0, 0.2, 0.4 and 0.6 mg L^−1^ Fe as FeSO_4_.7H_2_O and 0, 10, 20 and 30 mg L^−1^ Cd (supplied as CdSO_4_).
(PNG)

S2 Fig Experimental photograph. Mn and Cd indicates 0, 0.5, 1 and 1.5 mg L^−1^ Mn as MnSO4 and 0, 10, 20 and 30 mg L^−1^ Cd (supplied as CdSO_4_).
(PNG)
