# Supplementary figures and images for: Effects of Fe and Mn cations on Cd uptake by rice plant in hydroponic culture experiment
Source: PLoS One. 2020 Dec 10;15(12):e0243174. doi: 10.1371/journal.pone.0243174 (PMC7728178; doi:10.1371/journal.pone.0243174)

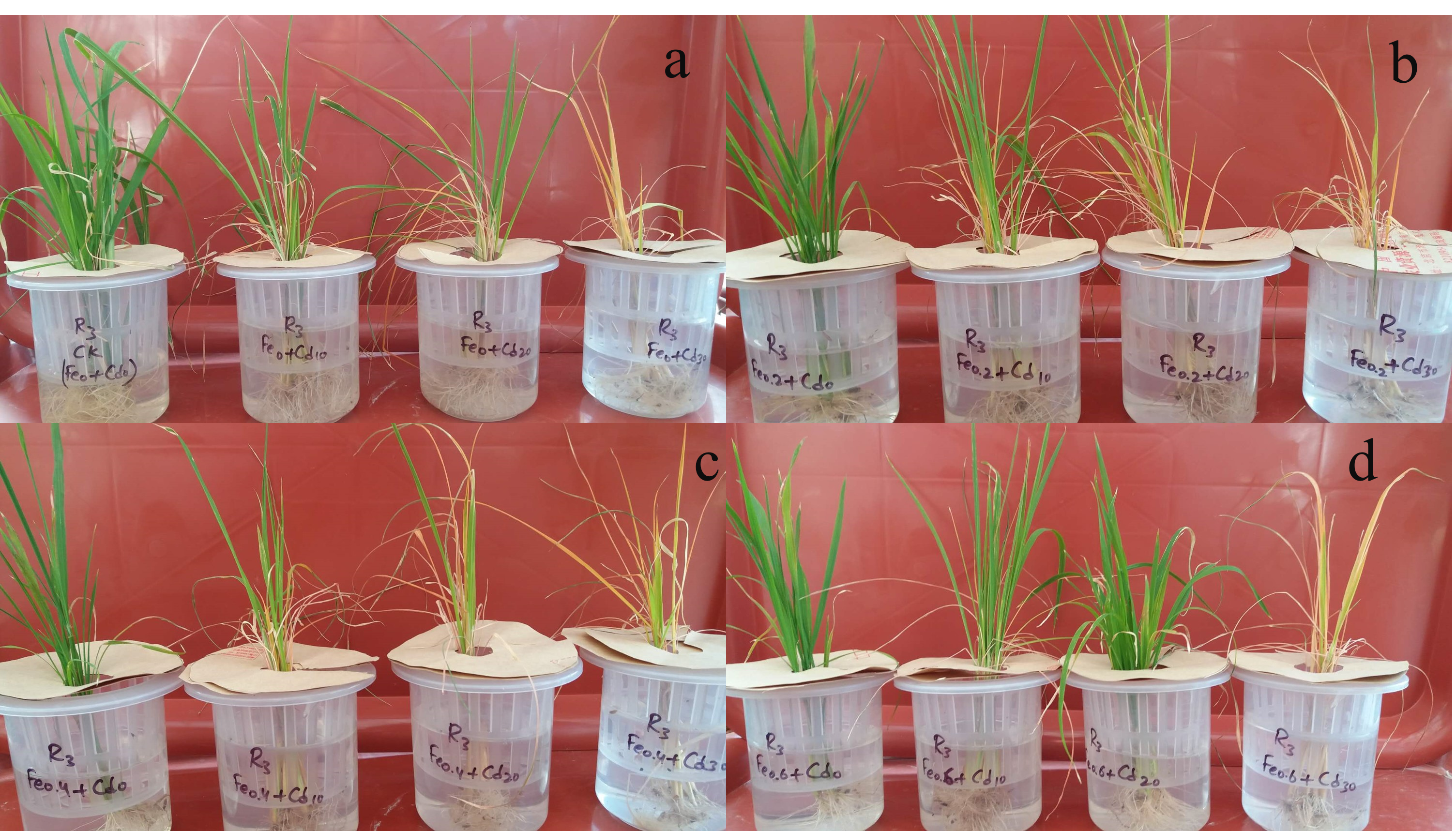

Supplement: S1 Fig — Fe and Cd indicates 0, 0.2, 0.4 and 0.6 mg L−1 Fe as FeSO4.7H2O and 0, 10, 20 and 30 mg L−1 Cd (supplied as CdSO4). (PNG) [file pone.0243174.s001.png]

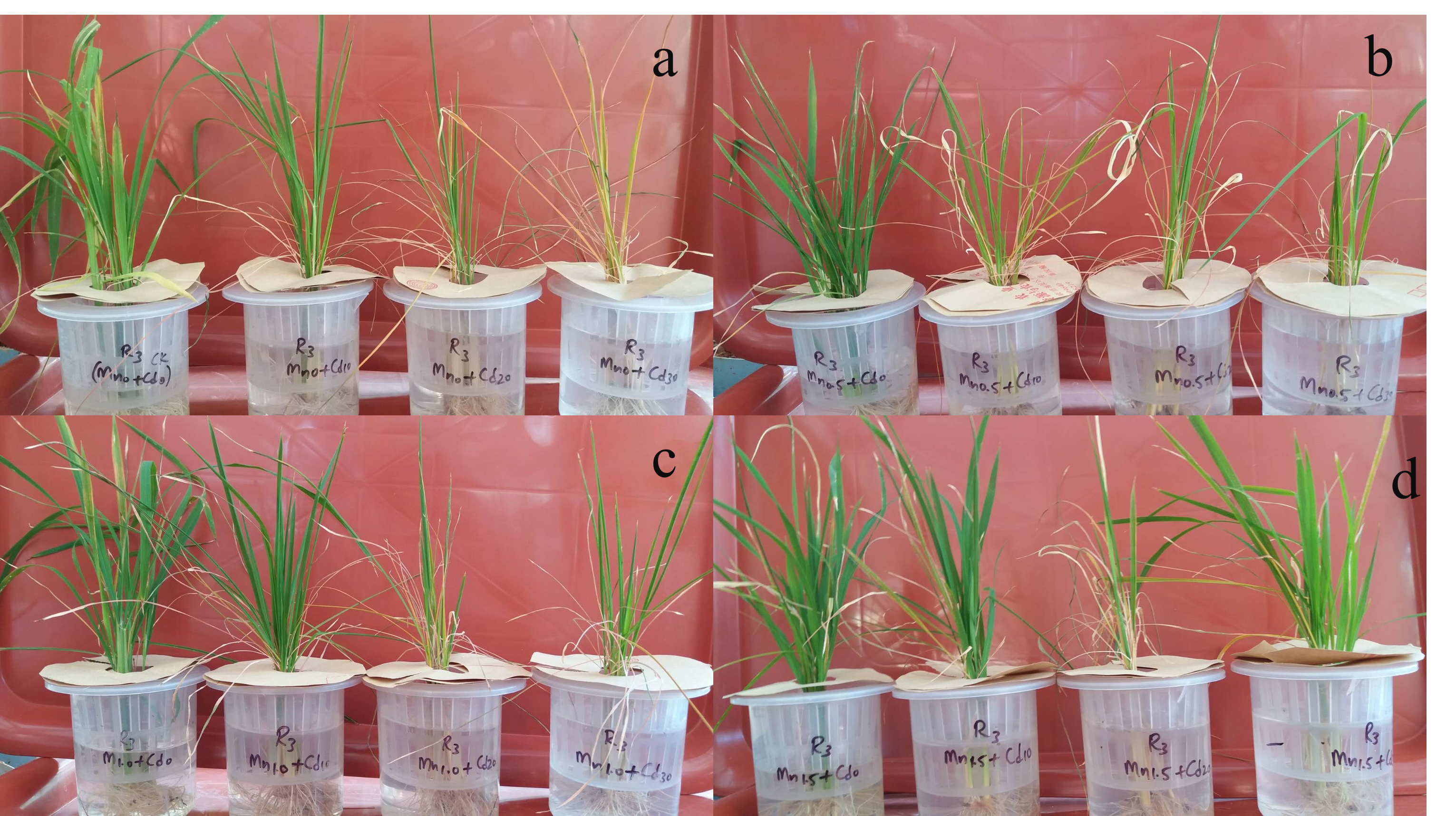

Supplement: S2 Fig — Mn and Cd indicates 0, 0.5, 1 and 1.5 mg L−1 Mn as MnSO4 and 0, 10, 20 and 30 mg L−1 Cd (supplied as CdSO4). (PNG) [file pone.0243174.s002.png]
